# Low-intensity pulsed ultrasound treatment improved the rate of autograft peripheral nerve regeneration in rat

**DOI:** 10.1038/srep22773

**Published:** 2016-04-22

**Authors:** Wenli Jiang, Yuexiang Wang, Jie Tang, Jiang Peng, Yu Wang, Quanyi Guo, Zhiyuan Guo, Pan Li, Bo Xiao, Jinxing Zhang

**Affiliations:** 1 Department of Ultrasound, Chinese People’s Liberation Army General Hospital, 28 Fuxing Road, Haidian District, Beijing 100853, China; 2 Department of Ultrasound, Beijing Hospital, 1 Dahua Road, Dongcheng District, Beijing 100730, China; 3 Orthopedics Research Institute of Chinese People’s Liberation Army, Chinese People’s Liberation Army General Hospital, 28 Fuxing Road, Haidian District, Beijing 100853,China

## Abstract

Low intensity pulsed ultrasound (LIPUS) has been widely used in clinic for the treatment of repairing pseudarthrosis, bone fractures and of healing in various soft tissues. Some reports indicated that LIPUS accelerated peripheral nerve regeneration including Schwann cells (SCs) and injured nerves. But little is known about its appropriate intensities on autograft nerves. This study was to investigate which intensity of LIPUS improved the regeneration of gold standard postsurgical nerves in experimental rat model. Sprague-Dawley rats were made into 10 mm right side sciatic nerve reversed autologous nerve transplantation and randomly treated with 250 mW/cm^2^, 500 mW/cm^2^ or 750 mW/cm^2^ LIPUS for 2–12 weeks after operation. Functional and pathological results showed that LIPUS of 250 mW/cm^2^ significantly induced faster rate of axonal regeneration. This suggested that autograft nerve regeneration was improved.

Nowadays, peripheral nerve injury is becoming the focus of social attention. Although autologous nerve transplantation is considered as the gold standard repair technique when primary suture was impossible^1^, the effect of autologous nerve transplantation is far from ideal^2^. Since 1940s, the recovery of its useful sensory function was not fully satisfied^3^. In 1993, Kallio demonstrated that the proportion of patients with S3 + level (on the Medical Research Council Classification Scale) recovery after autografting was 56% while it was 80% after direct suturing^4^. A meta-analysis including 384 nerve grafts reported that the proportion was 67%^5^. From the analysis of 1531 patients, Yang M *et al*.^6^ found only 49% of the patients had a meaningful recovery. Thus, improvement of the functional recovery after autologous nerve transplantation is of great significance.

In recent years, different methods have been used to improve peripheral nerve recovery, such as physical stimulus like laser^7^, electricity^8^, magnetic field^9^, shock waves^10^ and ultrasound^11^ have been examined. And promising results have been achieved^12^.

Much evidence showed that low intensity pulsed ultrasound (LIPUS) accelerated peripheral nerve regeneration, including SCs and injured nerves^13^,^14^,^15^. LIPUS was effective in these studies but the intensity was varied much^16^. LIPUS repetition pulse rates of 1.0–2.5MHz, intensities of 0.1–1.5 W/cm^2^ were referred in different reports^2^,^7^,^12^,^13^,^14^,^15^,^17^,^18^,^19^. Previous studies have employed different doses of ultrasound. Tsuang YH^2^ found that SCs showed a trend to survive without damage under an intensity of 300 mW/cm^2^ ultrasound. Lv *et al*.^19^ demonstrated that both gene and protein expressions were facilitated in neurons and SCs under an intensity of 500 mW/cm^2^. Chang applied a frequency of 1.5 MHz with an intensity of 300 mW/cm^2^ 14 ultrasound, and a frequency of 1 MHz with an intensity of 200 mW/cm^2^ ultrasound on sciatic nerve defects conduited rats for 12 ultrasonic treatment sessions over 2 weeks after 1 day of rest^18^. In order to define a more effective treatment option including frequency, intensity, length of time and cycle, we employed three different doses: low-dose (250 mW/cm^2^), mid-dose (500 mW/cm^2^) and high-dose (750 mW/cm^2^) of low-intensity ultrasound.

Nerve injury models used to explore the promoting regeneration effect on peripheral nerve were sciatic nerve crush model^17^, sciatic nerve neurotomy model^13^ and PLGA or silicon conduits interposed into sciatic nerve defect gaps model^18^ in mammals.

Previous LIPUS *in vivo* studies were in the peripheral nerve repairation after injuries, but few studies were in the nerve regeneration after the gold standard treatment-autologous nerve transplantation.

The purpose of this study is to explore the therapeutic effect of three different ultrasound intensities (250 mW/cm^2^, 500 mW/cm^2^ and 750 mW/cm^2^) on the grafts of autologous nerve reversal transplantation. The evaluation of target organs was usually by comparing weighing muscles and the cross-sectional areas of fibers. Here, we first apply contrast-enhanced ultrasonography (CEUS) for evaluating muscle functional recovery on the autograft animal models.

## Results

**Figure 1:**
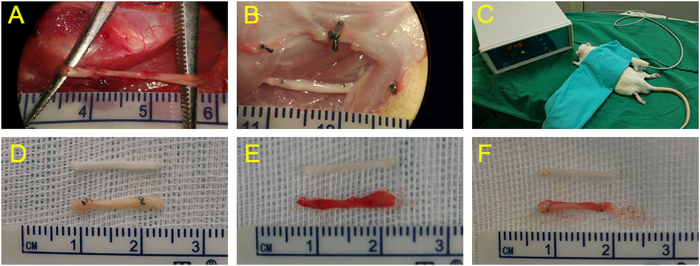
In the control group, 12 weeks after transplantation, an autograft sciatic nerve was 10-mmlong and swelling, adhesion to the surrounding tissue (**A**). In the low-dose group, an autograft sciatic nerve was 10-mmlong and with less nervous tissue inflammation (**B**). Experimental apparatus for applying low-intensity pulsed ultrasound. The transducer was placed on the right leg operational area skin via ultrasound gel (**C**). General view of different group sciatic nerve: low-dose group (**D**), high-dose group (**E**) and control group (**F**).

All the sciatic nerves were successfully reverse-autografted with the length of 10-mmlong. (Fig. 1).

### Sciatic nerve Functional analysis

**Figure 2:**
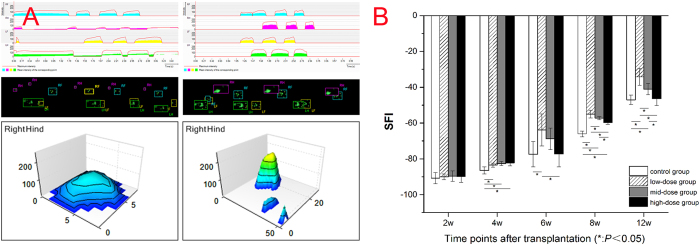
Functional analysis results. (**A**) 2-D toes force diagrams, footprints of rats and 3-D toes force diagrams of control group (left) and low-dose group (right). (**B**) Graphic plot of SFI behavior according to groups. SFI was gradually increased from the 2w after operation. SFI of the low-dose group increased most.

During 12 weeks, Catwalk automated gait analysis system clearly recorded the rat footprints of each group. 2-D toes force diagrams showed that all the rats’ maximum and mean stress intensities and the time contacting with the ground gradually increased over time. The changes of low-dose group were dramatic while no change of high-dose group was observed. The intensity and the time were optimal in mid-dose group compared to the control group. 3-D toes force diagrams showed that right hind feet’s stress focus moved back and stress area was increased. Besides in the high-dose group, the distance between the first and fifth toe (TS) and the distance between the second and fourth toe (ITS) of the operation side in other three groups were close to those in the contralateral side and the maximal distance between the tip of the longest toe and the heel (PL) had no considerable increasing. (Fig. 2A).

There was no significant difference of sciatic nerve index (SFI) among all the groups at 2w (*P* = 0.914). SFI of treatment groups was significantly higher than control group at 4w (*P* = 0.002) and 8w (*P* = 0.000). SFI of high-dose group has no significant difference compared to control group at 6w (*P* = 0.191) and 12w (*P* = 0.798) and was significantly lower than that of low-dose and mid-dose groups at 8w and 12w (*P* = 0.000). SFI of low-dose group was the highest among all the four groups beginning at 6w. (*P* = 0.026, *P* = 0.000, *P* = 0.000, Fig. 2B).

### Electrophysiological evaluation

**Figure 3:**
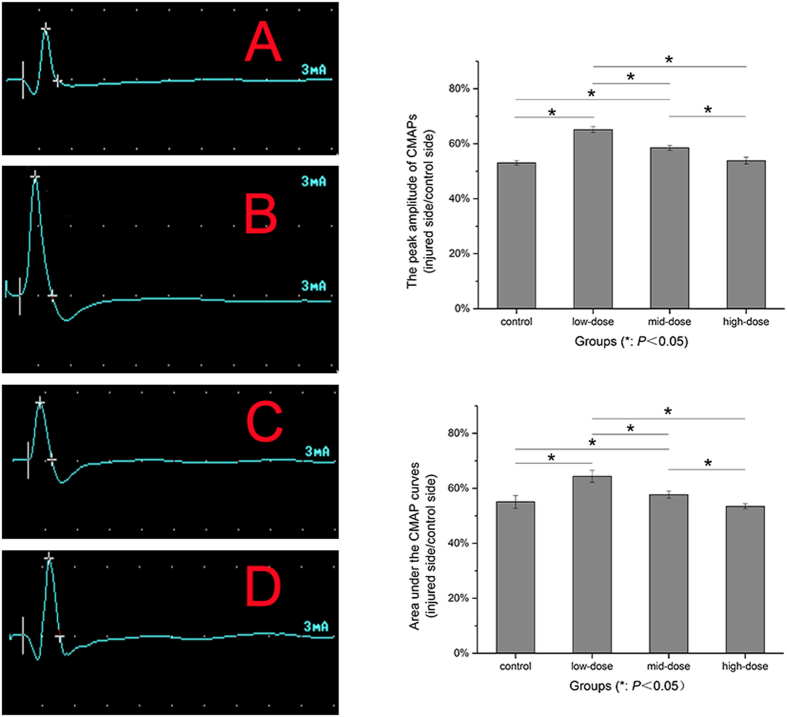
Recovery index of compound muscle action potential (CMAP) curves after 12-week operation. Besides there was no significant difference between recovery indexes in the high-dose group and the control group, there was significant difference between the indexes in the other two groups. (**A**) control group, (**B**) low-dose group, (**C**) mid-dose group, (**D**) high-dose group.

At the end of 3-monthsurgery, the recovery indexes of the low-dose and mid-dose treatment groups were significantly increased than the control group (*P* = 0.000), and the low-dose group was dramatic obvious. There was no significant difference between the high-dose group and the control group (*P* = 0.238). (Fig. 3).

### Evaluation of the axons growth

**Figure 4:**
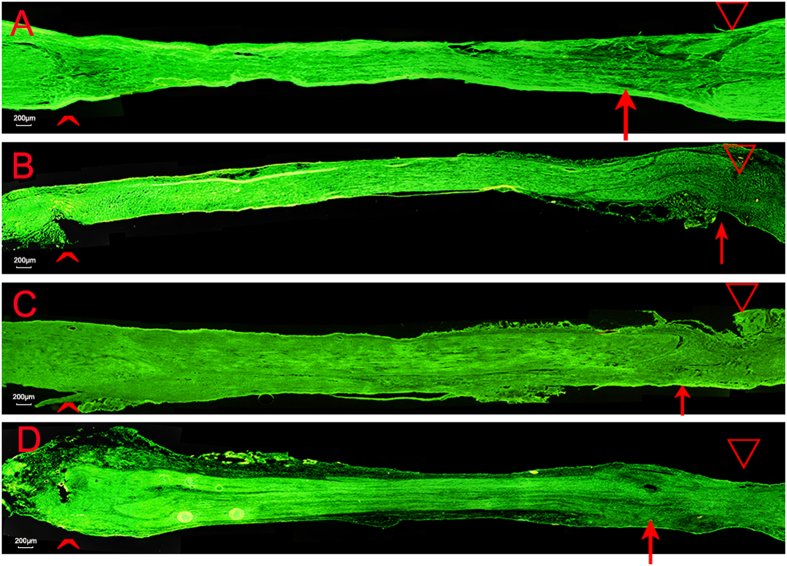
Longitudinal sections with NF200 staining of regenerated sciatic nerve tissue 8-week postopertaion in four groups. (**A**) control group, (**B**) low-dose group, (**C**) mid-dose group, (**D**) high-dose group. **^**: proximal end, ∇: distal end, ↑: end point of regenerated nerve axon.

The immunopositivity of NF200 proteins was used as markers of axons in the regenerating nerves at 8 weeks and 12 weeks. At 8 weeks, the lengths of the axon growth in three treatment groups were longer than the control group. The lengths of the axon growth of the low-dose group were longer than those of the other two treatment groups. The lengths of the mid-dose group were longer than those of the high-dose group. (Fig. 4). At the end of 12 weeks, there was no difference among all four groups.

**Figure 5:**
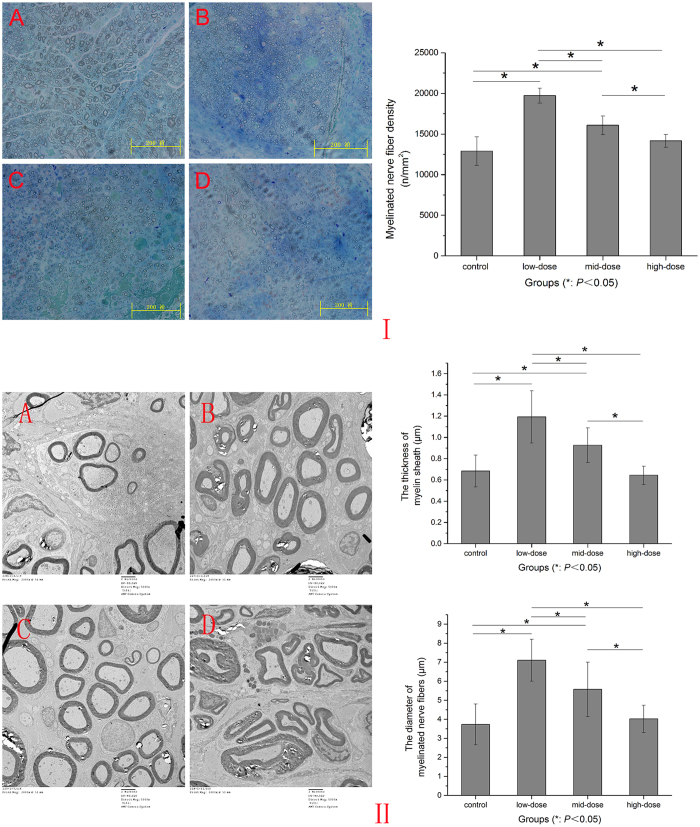
Evaluation of the axons growth. I: Toluidine blue staining at midpoints of the four groups autograft nerves. (**A**) control group, (**B**) low-dose group, (**C**) mid-dose group, (**D**) high-dose group. Myelinated nerve densities of four groups 12-week postoperation. II: Electron microscopic photographs of nerves in control (**A**), low-dose (**B**) mid-dose (**C**) and high-dose (**D**) groups 12 weeks after surgery. The thickness and diameter of axons of four groups 12-week postoperation.

Midpoint toluidine blue staining of the autograft nerves showed that every group had characteristic appearance of regenerated nerves. Low-dose group had slightly larger, more thickly myelinated axons than the other three groups. The control group had smaller and thinner myelinated axons than the mid-dose group and high-dose group. The regenerated myelinated nerve fiber densities of each group were: control group 12910.8 ± 1764.1395 (n/mm^2^), low-dose group 19725.6 ± 913.9044 (n/mm^2^), mid-dose group 16089 ± 1154.3745 (n/mm^2^) and high-dose group 14172.4 ± 813.7843 (n/mm^2^). Except that the first and the fourth groups had no significant difference (*P* = 0.121), the other groups had significant difference between each other (*P* = 0.000). (Fig. 5I).

Ultra-thin sections taken from four groups autograft sciatic nerve at 12-week postoperation observed under electron microscope revealed that the regenerated axons were observed in all groups. The thickness and diameter of axons in low-dose group were larger than those of other groups. Except that the first and the fourth group had no significant difference (*P* = 0.728) (*P* = 0.680), other groups had significant difference between each other (*P* = 0.000) (*P* = 0.001). (Fig. 5II).

### The wet weight gastrocnemius muscles

**Figure 6:**
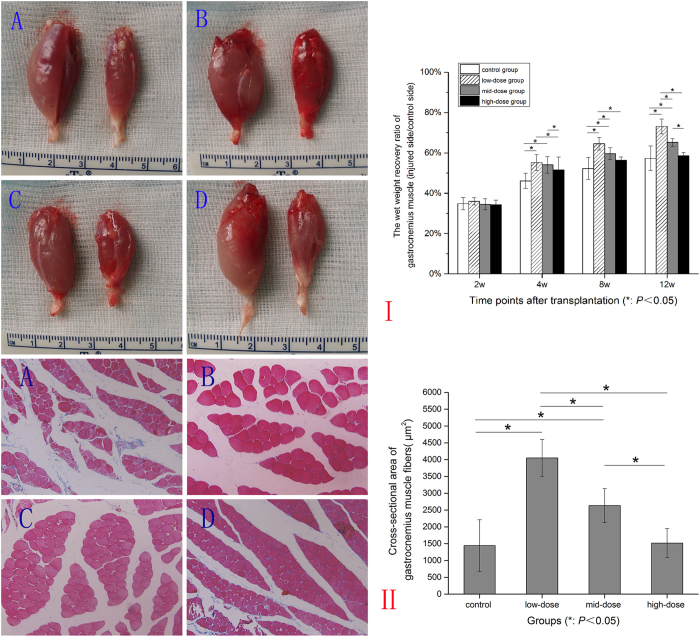
The wet weight gastrocnemius muscles. I: Contrast figure of gastrocnemius muscles in all groups. (**A**) control group, (**B**) low-dose group, (**C**) mid-dose group, (**D**) high-dose group. The contralateral side was on the left, the operational side was on the right side. The wet weight recovery ratio of gastrocnemius muscle (injured side/control side) of each group changed at different time points. II: Masson trichrome and cross-sectional area of gastrocnemius muscle in four groups. (**A**) control group, (**B**) low-dose group, (**C**) mid-dose group, (**D**) high-dose group.

In this study, gastrocnemius muscles in operation side atrophied seriously. The wet weight recovery ratio of gastrocnemius muscle of each group gradually increased from 2 weeks to 12 weeks after operation. Except for the ratio between the control group and the high-dose group (*P* = 0.725), the ratio of other groups had significant difference between each other, (*P* = 0.032) (*P* = 0.000) (*P* = 0.000). The low-dose group increased significantly. (Fig. 6I).

Masson trichrome of the cross-sectional gastrocnemius muscle showed that fibers in the low-dose group arranged regularly while control group and high-dose group arranged irregularly. And the area of fibers in low-dose group was the maximum (*P* = 0.000). (Fig. 6II).

### The blood perfusion of the gastrocnemius muscles

**Figure 7:**
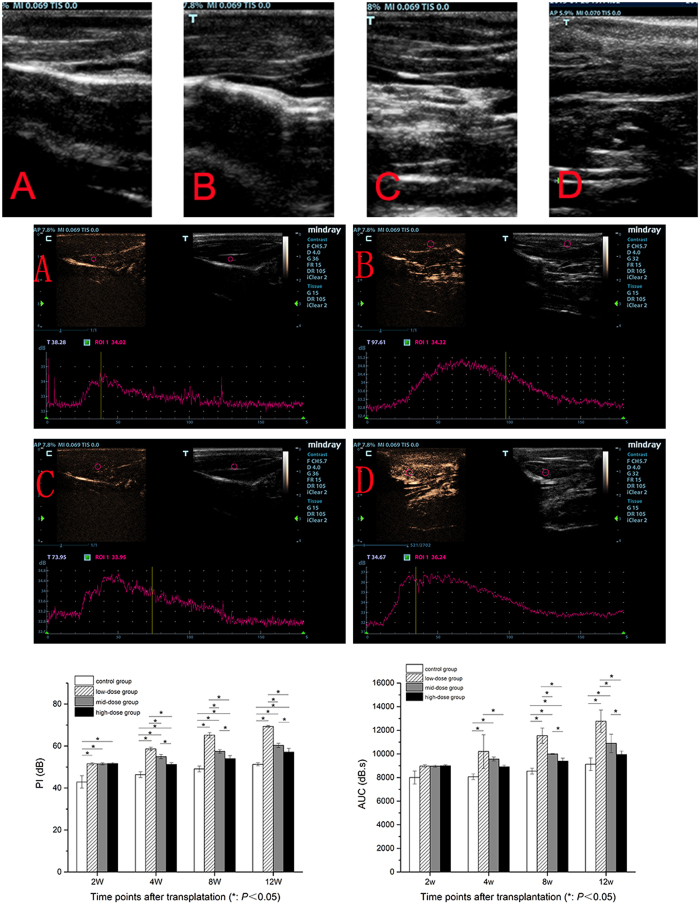
Gray-scale CEUS of four groups. (**A**) control group, (**B**) low-dose group, (**C**) mid-dose group, (**D**) high-dose group. 2-D images and time-intensity curves of all groups at the end of 12 weeks. PI and AUC of all groups at different time points.

Through the time-intensity curve, the peak intensity (PI) and the area under the time-intensity curve (AUC) of each group at different time points were obtained. The PI of the control group was lower than other three groups at all time points. The PI of the low-dose group was higher than other three groups and the PI of high-dose group was higher than that of the control group from 4 weeks after operation (*P* = 0.000). The AUC of low-dose group were higher than other groups from 4 weeks after operation (*P* = 0.002) (*P* = 0.000) (*P* = 0.000) and the AUC of high-dose group had no significant difference with that of the control group at the end of 12 weeks (*P* = 0.077). The PI and AUC of the mid-dose group were in the middle level among four groups all the time. (Fig. 7).

### Expression of NF-κB p65 protein

**Figure 8:**
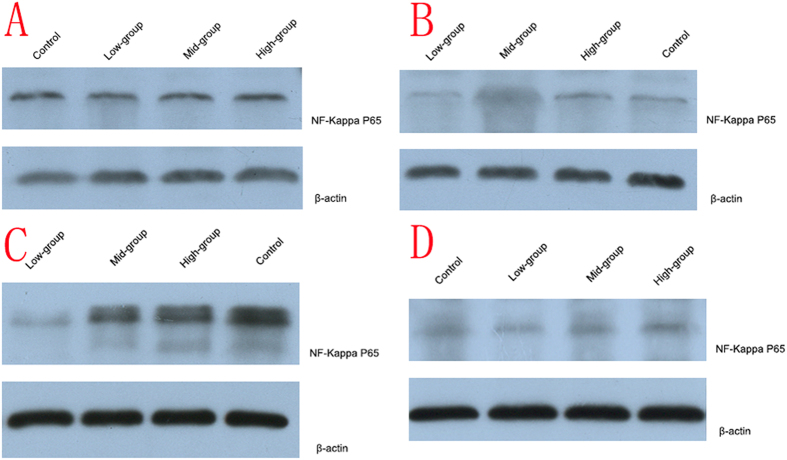
Expression of NF-κB p65 in four groups. (**A**) 2 weeks after operation, (**B**) 4 weeks after operation, (**C**) 8 weeks after operation, (**D**) 12 weeks after operation.

Western blotting (WB) was carried out. The expression of of NF-κB p65 protein was no significant difference between control group and high-dose group (*P* = 0.661), low-dose group and mid-dose group (*P* = 0.388) at 2 weeks after operation. Expression of NF-κB p65 protein in low-dose group was lower than other groups from 4 weeks after operation (*P* = 0.000). Protein expression of high-dose group was unstable and was no significant difference with control group at the end of 12 weeks (*P* = 0.122). Expression of NF-κB p65 in mid-dose group was higher than in low-dose group and lower than in control group (*P* = 0.000). (Fig. 8).

### The correlative analysis

At the end of 12 weeks after surgery, in all four groups (5 rats each group, n = 20), the PI and AUC were linearly positive correlated with recovery index of CMAP curves and were linearly negative correlated with WB 12-week postoperation (Table 1).

## Discussion

LIPUS has been proved to have therapeutic effect on a lot of tissue damage. LIPUS of 49.6 mW/cm^2^ and 57 mW/cm^2^ intensities, 1 MHz repetition pulse rate were initially used in the repairing of pseudarthrosis and bone fractures by Duarte in 1983^20^. The US Food and Drug Administration approved LIPUS for accelerating fresh fracture healing in 1994 and for the treatment of existing nonunions in 2000 separately^21^. Many reports confirmed that LIPUS intensities of ≤100 mW/cm^2^ could affect biological process such as skin healing of alloxanic diabetic rats (Nolasco 1993), repairation of chronic varicose ulcers (Hilario, 1993), chronic inflammatory process (Pires 1994) and healing of osteogenesis (Rubin,2001)^13^. LIPUS could also promote healing in various soft tissues such as cartilage, intervertebral disc, etc. It could improve bone-tendon junction through effect on osseous tissue^22^. In recent years, researchers also found that LIPUS could promote the differentiation of neurite outgrowth and neural stem/progenitor cells (NSPCs)^23^, induce pluripotent stem cells–derived neural crest stem cells (iPSCs–NCSCs)^19^, suppress adipogenesis and facilitate osteogenesis of mesenchyme stem/progenitor cell (MSC) lines^24^.

The ultrasonic effect on the nervous system was first concerned in the early stage of the ultrasonic biological effect^5^. In 1929, Harvey reported that gastrocnemius muscle of frog twitched when frog sciatic nerve was stimulated with ultrasound. In 1939, Pohlman and co-workers first reported that ultrasound could successfully treat sciatica and branchial plexus disorders. Scholtz and Stuhifuth separately confirmed that ultrasound played significant role in clinical effects with nervous tissue^5^. Then, many studies have been performed to identify the potential mechanisms. Those studies mainly concentrated on continuous-pulsed-ultrasound and the mechanisms were argued at thermal or non-thermal effects^25^. The ultrasound intensities of 0.05–0.5 W/cm^2^ are widely used as an imaging modality nowadays.

Previous studies demonstrated that the therapeutic effect of LIPUS on damaged rat sciatic nerve and explained the mechanism of LIPUS on peripheral nerve regeneration. Autologous nerve transplantation model was usually used as control group in peripheral nerve regeneration. Studies of LIPUS on promoting peripheral nerve stayed still in the fundamental research. However, patients were more concerned with the recovery of damaged nerve after operation.

Fox *et al*. demonstrated that nerve fiber density reaches a plateau at 3 months post-injury using different methods of nerve repair^26^. Nerve repair experiments in animals usually choose 4, 8, 12 week as the testing time points. Previous research showed that the SFI of ultrasound treated rats group was improved from the 14th day after the crush injury^12^. In our research, we added the 2-week time point to explore whether early changes would be detected.

We found that SFI and the recovery indexes (peak amplitude and area under the CMAP curves) of treatment groups were significantly higher than those of control group at 12 weeks. NF200 protein specific staining confirmed that the nerve regeneration speed of treatment groups were significantly faster than that of control group at 8 weeks. Apart from that, the target muscle wet weight, the pathological histology and blood perfusion of target muscles in treatment groups were also significantly differed from those of the control group. Our study confirmed that LIPUS could improve the recovery of nerve regeneration.

**Figure 9:**
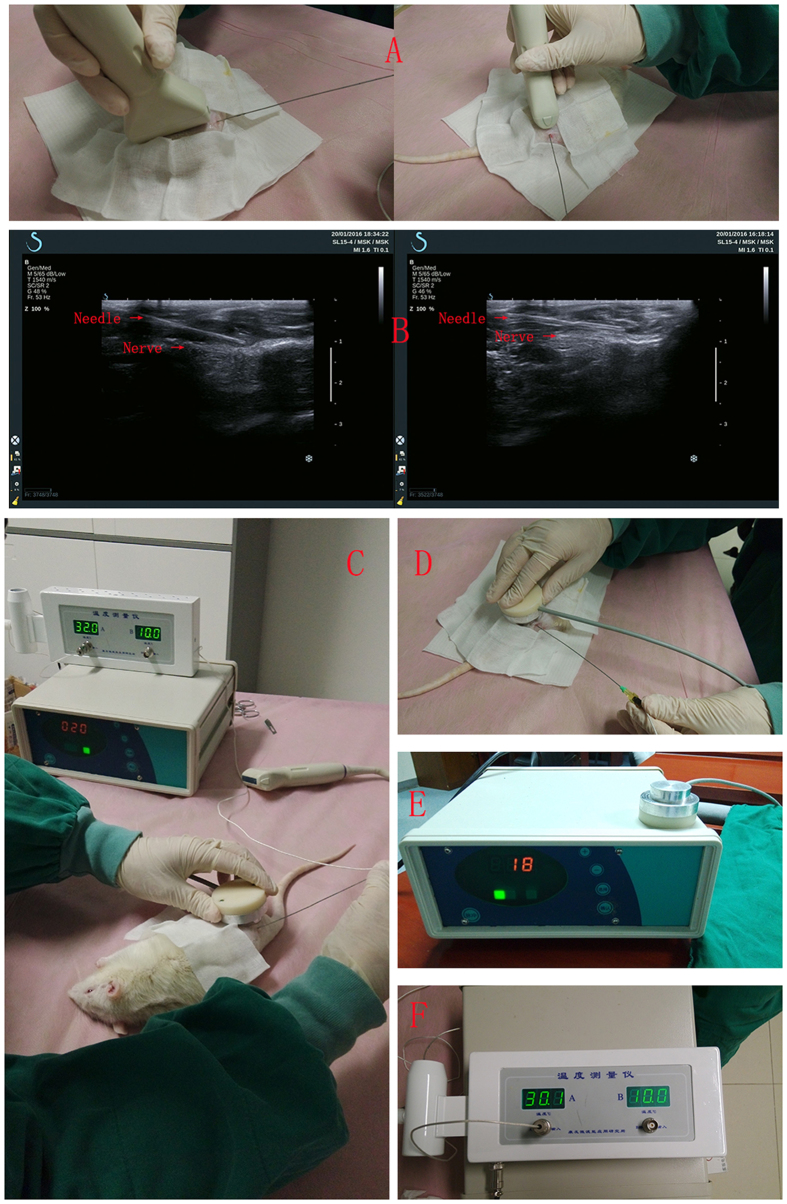
(**A**) The temperature measuring needle was placed into the tissue space near the sciatic nerve under ultrasound guided; (**B**) 2D-ultrasonic images showed the location of the temperature measuring needle; (**C**) Detection of the temperature during the process of treatment; (**D**) The positions of treatment probe and the temperature measuring needle; (**E**) An example of ultrasonic treatment apparatus display; (**F**) An example of temperature measurement instrumental display.

**Figure 10:**
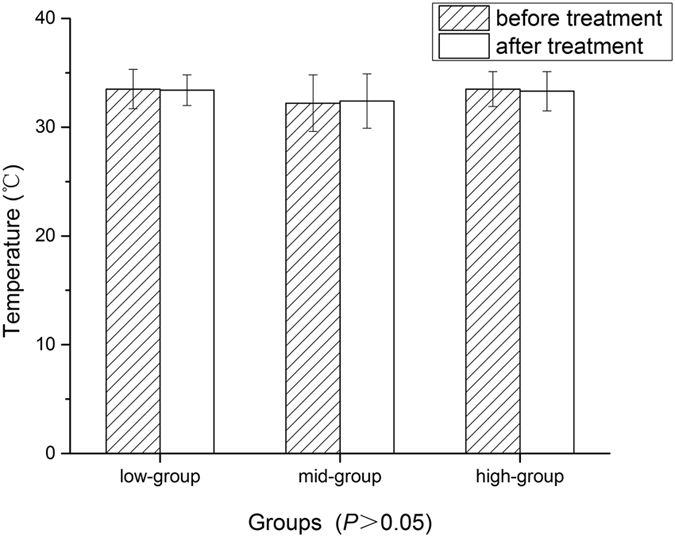
The comparison of temperature between three groups before and after the 5 min ultrasonic treatment.

In the present study, the effect of low-dose and mid-dose groups was superior to that of high-dose group. This is consistent with previous report^14^. Mechanical effect were recognized as main effect in the biologic action of LIPUS while thermal mechanisms were not^13^. The results from 30 rats evidenced that there is no change of local temperature before and after different intensity-ultrasonic treatment in our additional experiment (Table 2, Figs 9 and 10). Micromechanical stimulation and cavitation mechanisms played an important role^27^. Mechanical strains may result in increasing enzyme activity and modifying cell metabolism. Studies showed that LIPUS could promote the permeability and selectivity of cell membrane and cell wall, enhance mass transfer and increase absorbing of nutritional elements. Through this way, it could increase the survival and proliferation of seeded SCs^14^ which was key in peripheral nerve regeneration. However, excessive LIPUS could cause the worse curative. High-dose LIPUS could cause high transient intensity of acoustic pressures and high local temperature, which could destroy cell membrane, cytoskeleton, chloroplasts and mitochondria^28^. The microenvironment caused by high-dose LIPUS may be much suitable for formation of free radicals which could increase tissue inflammation reaction^29^. Our study was consistent with previous conclusions, and confirmed that SFI and morphological changes were worse in high-dose group than other three groups.

In this study, we also found lower intensity LIPUS had better effect, from the beginning time point to the end, than the higher intensity LIPUS. The effect of LIPUS on autograft sciatic nerve displayed at 4w after transplantation especially in low-dose group. Previous studies showed that the SFI of ultrasound treated rats group was improved from the 14^th^ day after the crush injury while that of the untreated control group was improved from 18^th^ day after the operation^12^,^17^. In our study, the time point was a little later compared these results. The possible reason was that autograft might spend more time on nerve regeneration after dissection trauma while crush injury nerve spend less time on nerve repairation. There was significant enhancement in low-dose group and in mid-dose group during the process of LIPUS treatment. While at the end of 12w, curative effect was only significant in low-dose group while was uncertainty in both mid-dose and high-dose groups. Our results revealed that low-dose LIPUS was effective persistently and constantly. Actually, it has been reported that 0.5 W/cm^2^ would be enough to accelerate regeneration of peripheral nerve and the damaged nerve was more sensitive to ultrasound than intact nerve^12^. We reckoned that low-dose LIPUS could provide appropriate mechanical signal to promote local new blood vessel formation, improve nerve sprouting stimulation and release more nutritional chemical mediators. However, mid-dose and high-dose LIPUS treatment could only induced part of these effects.

Muscle atrophy evaluation is used to assess the nerve generation indirectly. Muscle wet weight, histopathologic examinations of the affected muscle are also included in conventional methods. While these methods are invasive and can only be performed in animal studies. In this study, we first used CEUS to evaluate the perfusion of the denervated muscle. CEUS is an noninvasive method for the assessment of the tissue perfusion and has been widely used to evaluate the perfusion of myocardium, kidneys, peripheral nerve and other organs^30^. It uses microbubbles (<10 μm in diameter) as contrast agent and utilizes backscattering theory for contrast sonography. The more bubbles, the higher ultrasonic intensity. The microbubbles’ behavior is similar to red blood cells in the body circulation especially in blood capillary, but they cannot extravasate into the interstitial space and eventually be metabolized to gas dispelling in the body. Previous studies have showed that there is a positive correlation among PI, AUC and microbubbles numbers. So PI and AUC can be measured for tissue perfusion. In this study, it indicates that the higher PI and AUC, the better muscle function recovery. PI and AUC of low-dose group were higher than those of other groups, those of mid-dose group were higher than control group. While those of high-dose group and control group had no significant difference.

In this study, we also found that PI and AUC in all groups were positively correlated with the recovery indexes (peak amplitude and area under the CMAP curves of the operational side divided by those of the nonoperational side). PI and AUC in all groups were also negatively correlated with brightness of NF-κB p65 expression by western blot analysis. Zhao H *et al*.^31^ confirmed that VEGFR-2 signaling was essentially in early recovery phase of muscle denervation. As we know, VEGF signaling through VEGFR-2 is the major pathway activating angiogenesis. LIPUS treatment can improve the regeneration of nerve fibers reaching the target muscles, so it can prevent the irreversible muscle atrophy. So we speculated that once the target muscles recontrolled by the regeneration nerve, the VEGF signaling would increase muscle blood perfusion. Thus, PI and AUC, indexes reflecting muscle perfusion, could directly respond the process. The correlation of PI, AUC, the recovery indexes and expression of NF-κB p65 were in accordance with our treatment effect. These outcomes suggested that CEUS might be used as a non-invasive method to evaluate the recovery of the denervated muscle.

This study had several limitations. First, the length of nerve defect was short. It was difficult to make a much longer nerve defecting model on a short sciatic nerve of Sprague Dawley rat. Second, the samples were limited in the study was. More rats were required in further experiments. Thirdly, the doses applied on rats in this study are not recommended for patients in autograft peripheral nerve regeneration. Further investigation was required to identify more accurate, constant and reproducible result prior to the clinical use.

Our study indicated that low-dose LIPUS might prompt a faster regeneration of the autografting sciatic nerve in the rat model. Further clinical studies will focus on the verification of the therapeutic effect on patients with a reversed autograft.

## Methods

### Animals

80 adult male Sprague Dawley rats, 5–6 weeks aged, weight 150–200 g, were used in this study. The animals were obtained from the animal center of the people’s liberation army military academy of medical sciences, Beijing, China. The experimental protocol was approved by the Ethics Committee of Chinese PLA General Hospital (2015-x10-02). All experiments were performed in accordance with the Revised Guide for the Care and Use of Laboratory Animals^32^.

### Experimental groups and surgical methods *in vivo*

80 SD rats were randomly divided into four groups: the control group (n = 20): autografting without ultrasonic treatment, low-dose group (n = 20): autografting with low-dose LIPUS treatment, mid-dose group (n = 20): autografting with mid-dose LIPUS treatment and high-dose group (n = 20): autografting with high-dose LIPUS treatment. Rats were anesthetized by 40 mg.kg^−1^ injection of 2% sodium pentobarbital into abdominal cavity.

All the rats received sciatic nerve autograft reversal transplantation surgery on the right hind legs. A 10-mm-long sciatic nerve was excised and then grafted in the reverse direction between the two nerve stumps using 9-0 Ethilon (Ethicon Inc., Sommerville, NJ, USA) sutures on either side to manufacture a 10-mm-long sciatic nerve autograft models. Muscle and skin were closed with 3-0 Ethilon sutures. All animals were raised in cages after operation.

5 rats of each group were sacrificed at week 2, 4, 8 and 12 after surgery for electrophysiological evaluation, ultrasonic testing, molecular biological detection and pathology detection.

### LIPUS treatment project

On the third day after surgery, the LIPUS treatment with the different doses was started to stimulate the sciatic nerves with an ultrasonic therapeutic apparatus (designed and manufactured by medical ultrasound engineering institute of Chongqing medical university, China). Ultrasonic probe diameter: 2.5 cm, acoustic frequency 1 MHz, duty cycle: 20%, pulsed repetition frequency 1 KHz. Acoustic intensity (spatial average temporal average, SATA): low-dose group 250 mW/cm^2^, mid-dose group 500 mW/cm^2^, high-dose group 750 mW/cm^2^. The ultrasonic therapeutic apparatus sets 1–20 different intensity grades. Grade 5, grade 13 and grade 18 represent the intensities of 250, 500 and 750 mW/cm^2^ ultrasound output, respectively. Total treatment time: 5 min per time, once every other day, until death.

### Functional analysis

Functional analysis was performed with the Catwalk automated gait analysis system (Cat Walk XT 10.5, Nolddus technology, Netherlands). The apparatus captures footprints through footprints light refraction technology. When a rat traverses the glass floor from one side to the other, high-speed camera under the walkway will record the real rat paws which are contacted with the glass floor. The following parameters were assessed: TS (in mm), ITS (in mm), PL (in mm). SFI will be automatically calculated by the system^12^.

### Muscle perfusion evaluation

The perfusion of triceps surae was evaluated by gray scale CEUS. CEUS was performed by using the M9 ultrasonic diagnosis instrument (Mindray Medical International Ltd) with an L12-5 transducer (5–12 MHz). The ultrasonography contrast agent was SonoVue^®^ (0.025 ml.kg-1; Bracco, Milan, Italy) and it can provide 8 μl/ml of sulphur hexafluoride microbubbles (SF6) after reconstitution with 5 ml of saline. It was administered with a quick bolus through the tail vein. The scans were preserved from the injecting moment to three minutes after injection. The ultrasound scan parameters, such as gain, scanning depth and time gain control, were optimized and remained constant throughout the whole study. The dynamic CEUS images were recorded as cine-loop for offline analysis. The muscle perfusion was analyzed by quantitative analysis sofeware (M9, Mindray Medical International Ltd). The area of ROI was a circle of 30–40 mm^2^ area, and the ROI was tried to avoid large vessels^33^. Parameters included the PI and the AUC.

### Electrophysiological evaluation

At the end of 12 weeks after surgery, under anesthesia, the both rat sciatic nerves and posterior gastrocnemius muscle were carefully re-exposed and dissected from surrounding tissues. Stimulating needle electrodes connected to an electrical stimulator (Keypoint; Medtronic company, Denmark) were placed on the proximal and distal autograft nerve or normal nerve. Recording needle electrode was placed in the distal gastrocnemius muscle of the same side. Ground electrode was also placed in subcutaneous tissue upper extremity. The nerve stimulation parameters used were 1 Hz pulse and 3 mA current. Compound muscle action potential (CMAP) of all groups was recorded. CMAP peak amplitude and area under the CMAP curve were analyzed offline^7^. The recovery index was calculated as the peak amplitude and the area under the CMAP curves of the operational side divided by those of the nonoperational side^34^.

### Evaluation of growth axons

After the animals were sacrificed, the autograft sciatic nerve was harvested. In order to ensure the integrity of the autograft nerve, normal nerves near the proximal and distal suture points should be separated. Then nerves were frozen sliced longitudinally into 7-μm-thick samples. These samples were incubated in solution of anti-NF200 antibody (1:200; Sigma) overnight at 4 °C temperature. After washed three times with PBS, they were incubated in biotinylated anti-mouse goat IgG/Alexa Fluor^®^ 488 solution (1: 50) for 2 hour. Washed three times with PBS and used blue fluorescence under the fluorescent microscope to observe autograft nerve axons regeneration.

A 1.5-mm segment of the midpoint of the autograft nerve was dissected. The samples were fixed in 2.5% glutaraldehyde for 6 hours, washed three times with PBS, fixed in 1% osmic acid for 2 hours, washed three times with PBS, dehydrated, and plastic embedded. Transverse sections of semi-thin (0.7 μm) and ultra-thin (70 nm) sections were obtained by an ultramicrotome (Leica EMUC-7, Wetzlar, Germany). The semi-thin sections were stained with 1% toluidine blue for 10 min and observed under light microscopy and photographed. Total number of nerve fibers was obtained by Image Pro Plus (Media Cybernetic, Bethesda, MD, USA). The ultra-thin sections were stained with 3% uranium acetate-lead citrate and observed under CM-120 transmission electron microscope (Amsterdam, Netherlands) and photographed. The myelinated nerve fiber diameter and thickness of myelin sheath were analyzed by Image Pro Plus.

### Weighing triceps surae muscles and Masson trichrome staining

Isolated the operational and contralateral side triceps surae muscles and cleaned the surface blood with physiological saline, dried with filter paper, weighed and recorded their weights by electronic balance scale (MS260, Shanghai, China). Specimens were fixed in 4% paraformaldehyde and paraffin embedded after dehydration, then cross-sectional cut into 5 μm thick slices in the triceps surae muscle belly and stained with Masson trichrome to measure the cross-sectional areas of the muscle fibers. The data was measured by use of Image Pro Plus. The wet weight recovery ratio of gastrocnemius muscle was calculated as the wet weight of gastrocnemius muscle of the operational side divided by that of the nonoperational side.

### Western blotting detection for NF-κB p65 expression of gastrocnemius muscle

Muscle tissues were collected and lysed with RIPA buffer and protease inhibitor. Tissue lysates (10 μg) were separated on a 10% SDS-PAGE gel and transferred to PVDF membranes and blocked with 5% nonfat milk (dissolved in 0.5%TBST) for 1 h at room temperature and then incubated overnight at 4 °C with primary antibody (1:500) : phospho p65 NF-κB. After washing in TBST, blots were incubated with second antibody (1: 3000) at 37 °C for 30 min in TBST. Finally, the membrane were washed three times with TBST for 15 min and the proteins were visualized using chemiluminescent substrate ECLA and ECLB (Healthcare, Bucks, UK) under fluorescence. The level of cellular actin was used as a loading control. (Table 3.)

### Statistical analysis

All variables were presented as mean ± standard variation. The data were analyzed by SPSS 17.0 (SPSS Inc., Chicago, IL). Statistical comparison between the groups was conducted using one-way analysis of variance. A Student-Newman-Kewls (SNK) post-test was performed to compare all pairs of groups. Correlation analysis use Pearson’s correlating analysis. The alpha level for all tests was 0.05. *P*<0.05 was considered statistically significant.

## Additional Information

**How to cite this article**: Jiang, W. *et al*. Low-intensity pulsed ultrasound treatment improved the rate of autograft peripheral nerve regeneration in rat. *Sci. Rep.* **6**, 22773; doi: 10.1038/srep22773 (2016).

## Figures and Tables

**Table 1:** The correlation between PI/AUC and the recovery index of CMAP curves/WB.

a			
Correlation coefficient	PI/peak amplitudeof CMAP	PI/area underCMAP curve	PI/ WB
*r*	0.84	0.73	−0.86
n	20	20	20
*P*-value	0.000	0.000	0.000
**b**			
**Correlation coefficient**	**AUC/peakamplitude of CMAP**	**AUC/area underCMAP curve**	**AUC/ WB**
r	0.89	0.77	−0.79
n	20	20	20
*P*-value	0.000	0.000	0.000

**Table 2:**
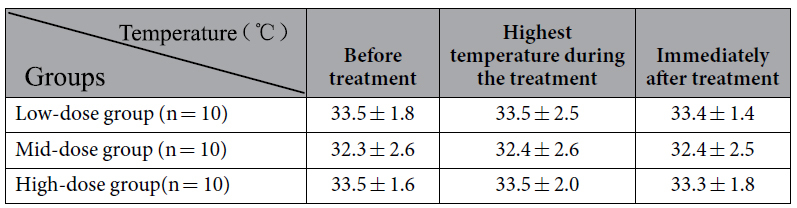
Data of temperature change during ultrasonic treatment in three groups.

**Table 3:**
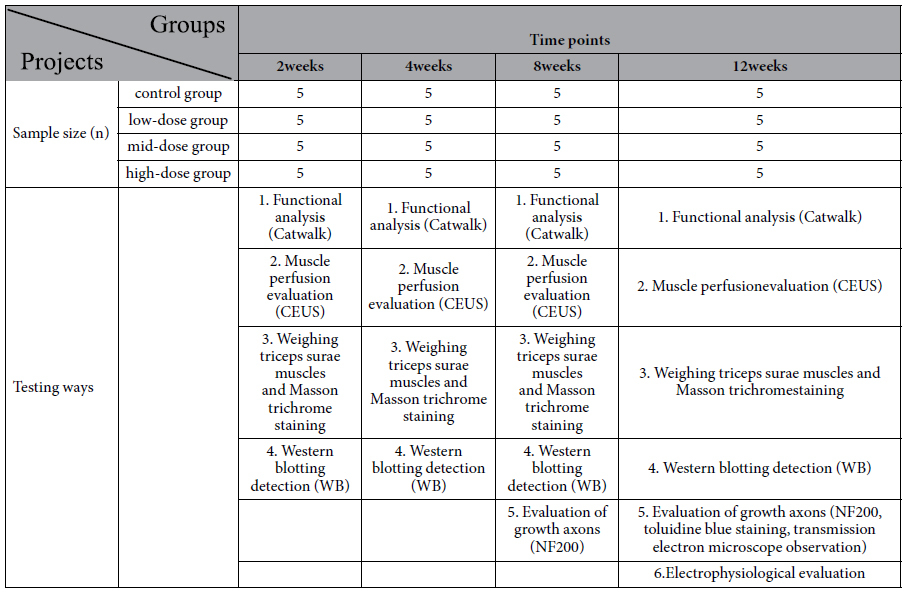
Study design presentation.
